# Seroprevalence of bovine leukemia virus and association with bovine infectious abortion in Creole breeds from tropical grazing herds in the Colombian Caribbean

**DOI:** 10.14202/vetworld.2024.1715-1721

**Published:** 2024-08-04

**Authors:** Misael Oviedo-Pastrana, Matiluz Doria-Ramos, Salim Mattar, Teresa Oviedo-Socarras, Darío Vallejo-Timarán

**Affiliations:** 1 Colombian Agricultural Research Corporation – AGROSAVIA, Turipana Research Center, Montería, Colombia; 2 University of Córdoba, Institute of Biological Research of the Tropics, Montería, Colombia; 3 University of Córdoba, Veterinary Medicine and Animal Sciences Faculty, Department of Livestock Sciences, Montería, Colombia; 4 Colombian Agricultural Research Corporation – AGROSAVIA, Obonuco Research Center, Pasto, Colombia

**Keywords:** bovine leukemia virus, co-infection, Creole breeds, infectious abortion

## Abstract

**Background and Aim:**

In the Caribbean region of Colombia, the concomitance of endemic infectious agents is a common problem, and coinfections are possible, increasing the complexity of cattle herds’ sanitary, reproductive, and productive problems. This study aimed to estimate the seroprevalence of bovine leukemia virus and its association with bovine infectious abortion in grazing Creole breeds from tropical herds in the Colombian Caribbean.

**Materials and Methods:**

For the determination of bovine leukemia virus (BLV), bovine viral diarrhea virus (BVDV), bovine herpes virus-1 (BoHV-1), and *Neospora Caninum* (NC), the enzyme-linked immunosorbent assay technique was used. Matrix analysis was performed to represent multiple seroprevalence in the same cow. To explore the association between the seroprevalence of BLV and bovine infectious abortion agents, a multivariate logistic regression model was used.

**Results:**

The seroprevalence was as follows: BLV 30.78%, BVDV 33.01%, BoHV-1 12.85%, and NC 8.96%. In the multivariate logistic regression model, seroprevalence of BVDV (OR 10.8; 95% CI: 7.5–15.6) and seroprevalence of BoHV-1 (OR 1.8; 95% CI: 1.1–3.0) were associated with the seroprevalence of BLV.

**Conclusion:**

Animals infected with BLV are more susceptible to coinfections with BVDV and BoHV-1. Implementing healthy measures against these two immunosuppressive infections could enhance the hygiene of numerous cattle herds. This study was designed as a retrospective cross-sectional study, which limits the ability to confirm that BLV is the primary infection. Further studies to confirm the primary infection of BLV with an active viral coinfection are necessary and the factors associated with these phenomena.

## Introduction

Bovine leukemia virus (BLV) is the most crucial neoplastic disease in cattle and is caused by a delta retrovirus of the *Retroviridae* family [1]. Research suggests that BLV carries a zoonotic risk and may contribute to human breast cancer [2]. In cattle herds globally, the infection results in substantial revenue losses and international trade complications [1, 3]. BLV’s underestimated economic impact results mainly from its asymptomatic nature and long clinical course as a chronic disease with minimal mortality [4]. The primary source of economic losses is decreased milk production, reduced weight gain, and culling. The immunosuppressive effect of BLV further increases the risk of acquiring secondary infections [5]. The silent nature of the infection, lack of a vaccine, and unavailable drug treatment make the control of BLV challenging. At present, the most controlled alternative against BLV is based on the application of diagnostic tests and elimination of positive animals; however, this strategy may not be economically viable in herds with a high prevalence of disease [1].

The cattle breeds Romosinuano (ROMO) and Costeño con Cuernos (CCC) are native to the Colombian Caribbean. The AGROSAVIA Turipaná Research Center is the leading conservation center in Colombia for these breeds. The Research Center is currently studying BLV, bovine viral diarrhea virus (BVDV), bovine α-herpesvirus-1 (BoHV-1), and *Neospora caninum* (NC) in ROMO and CCC breeds. The viruses BVDV, BoHV-1, and the protozoan NC are critical infectious agents related to abortion, infertility, and reproductive failure. In addition, BLV and BVDV have been recognized as important infectious agents that cause immune system dysfunction, affecting innate and adaptive immunity and predisposing animals to coinfections, increasing the severity of infections [5, 6]. In the Caribbean region of Colombia, BLV, BVDV, BoHV-1, and NC are endemic infectious agents, and coinfections are possible, increasing the complexity of cattle herds’ sanitary and productive problems.

Due to the importance of the production and conservation of Creole breeds for increasing the productivity of beef cattle herds under tropical conditions, research that contributes to increasing the knowledge about the relationship between BLV and other infectious agents is necessary to support the implementation of integral health management protocols, reduce the incidence of diseases, and improve the productivity of tropical beef cattle herds. This study aimed to estimate the seroprevalence of BLV and its association with bovine infectious abortion in grazing Creole breeds from tropical herds in the Colombian Caribbean.

## Materials and Methods

### Ethical approval

All animal studies were conducted in compliance with experimental practices approved by the Cooperative Education and Internship Association (Ethics Committee for AGROSAVIA Research – Approval no. 1828-2016). All cows were managed according to standard procedures to avoid or minimize any adverse effects on animal welfare.

### Study period and location

A retrospective study from July to December 2023 was conducted from a bovine blood serum bank collected in the year 2016 and stored at –80°C for subsequent serological analysis. The Cows belong to the Colombian germplasm bank program in the central nucleus of genetic conservation of the creole bovine breeds of the Colombian Caribbean, located in the Colombian Corporation of Agriculture Research (AGROSAVIA) - Turipaná Research Center, Montería City, Cordoba Region, Colombia. (N: 8° 50’ 58” and W: 75° 48’ 56”).

### Population and experimental design

This retrospective cross-sectional study was conducted on bovine Creole breeds (n = 848) from two tropical herds: An animal germplasm bank herd (AGB) and a genetic management program herd (GIP). Both herds belong to the program of germplasm banks in the central nucleus of genetic conservation of the Creole bovine breeds of the Colombian Caribbean, located in the Turipaná Research Center – AGROSAVIA, Monteria city from Córdoba region, Colombia.

### Sample size and sampling

Sample size calculation was performed assuming a closed population to estimate a single proportion [7]. The sample size was estimated with a confidence interval of 99%, a population size of 1500 animals (for finite population correction factor) and the hypothetical proportion of outcome factor (BLV) in a population of 62% [8], and a confidence limit of 100 (absolute ± %) of 3%. Using Open-Epi software [9], the minimum number of cows by the group was estimated to be 806. The sample size was adjusted for potential missing values, increasing the estimated sample size by 5%. The final number of cows was calculated from 848 animals. By simple random sampling, 403 animals of the ROMO breed and 445 of the CCC breed were selected.

### Cow sampling and data collection

A competitive enzyme-linked immunosorbent assay (ELISA) technique was used for BLV (INgezim BLV Compac 2.0, Gold Standard Diagnostics, Madrid, Spain) BVDV (INgezim IBR Compac 2.0, Gold Standard Diagnostics), and BoHV-1 (INgezim BVD DAS, Gold Standard Diagnostics) determination. The samples were considered positive or negative according to the absorbance value, and the selected cutoff points were Pos <0.54, Neg ≥0.84 for BLV; Pos <0.64, Neg ≥0.73 for BVDV; and Pos <0.22, Neg ≥0.75 for BoHV-1. An indirect ELISA technique (INgezim Neospora 3.0, Gold Standard Diagnostics,) was used to diagnose NC. For interpretation, the positivity index (IRPC) was calculated. Samples with IRPC ≥20 were considered positive, and samples with IRPC <20 were considered negative.

### Statistical analysis

The dataset includes herd variables (GIP or AGB); cow variables: Gender (male/female), age (≤1 year />1 year), breed (ROMO or CCC); and serological variables: Positive BLV, BVDV, BoHV-1, NC as categorical variables (yes/no). The seroprevalence for BLV, BVDV, BoHV-1, and NC was calculated as the number of cows with the condition divided by the number of cows enrolled in the study. A detailed matrix analysis was performed to represent multiple seroprevalence in the same cow.

To explore the association between the seroprevalence of BLV and bovine infectious abortion agents (BVDV, BoHV-1, NC), a bivariate analysis was performed using logistic regression analysis between each independent variable with BLV as the dependent variable. Variables were selected for the multivariate analysis with a value of p = 0.15 in the bivariate analysis. Variables that met the p-value criterion in the bivariate analysis were eligible for entry into a multivariate logistic regression model (p < 0.05) using backward selection. The model considered herd and cow variables as potential confounding variables. A mixed-effects logistic regression model was explored to establish a herd random effect. The odds ratio (OR) was used as a measure of association in the model. The logistic regression model’s goodness-of-fit was determined using Hosmer and Lemeshow’s test and normal residual analysis. All analyses were conducted using Stata^®^ Statistical Software version 18.0 (Stata Corp. Lakeway College Station, Texas, USA).

## Results

**Figure-1:**
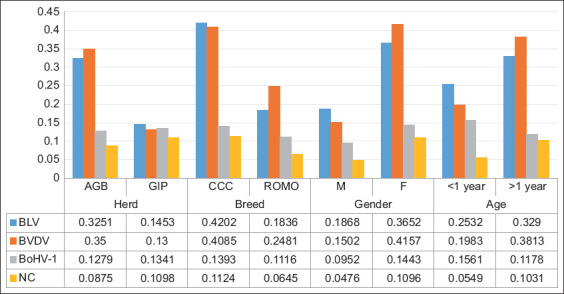
Distribution or infectious agents seroprevalence according to herd and cow variables in grazing Creole cows (n = 848) from two tropical herds in the Colombian Caribbean. BLV=Bovine leukemia virus, BVDV=Bovine viral diarrhea virus, BoHV-1=Bovine α-herpesvirus-1, NC=*Neospora caninum*, GIP=Genetic improvement program herd, AGB=Animal germplasm bank herd, CCC=Costeño con cuernos, ROMO=Romosinuano, M=Male, F=Female.

Of the 848 animals evaluated, 273 were males and 575 were females. ROMO was 403, and CCC was 445. The mean age was 3.4 years. Eighty-two animals belonged to the genetic improvement program (GIP) cattle herd and 766 to the AGB cattle herd. The seroprevalence for infectious agents evaluated was as follows: BLV 30.78% (n = 261), BVDV 33.01% (n = 280), BoHV-1 12.85% (n = 109), and NC 8.96% (n = 76). Figure-1 shows the distribution or seroprevalence according to the variables herd, breed, gender, and sex. The study found multiple seropositivity in the same cow; Table-1 shows the matrix of seropositivity among the infectious agents studied. It was observed that 17.4%, 19.3, 6.4, and 2.2 of the animals were seropositive for a single, two, three, or four infectious agents, respectively.

**Table-1:** Multiple seropositivity matrix in grazing Creole cows (n=848) from two tropical herds in the Colombian Caribbean.

Infectious agent	I	II			III			IV
			
	None	BVDV	BoHV-1	NC	BVDV BoHV-1	BVDV NC	BoHV-1 NC	BVDV BoHV-1 NC
BLV	59	123	8	4	30	13	5	19
BVDV	68	0	14	3	0	0	10	0
BoHV-1	11	0	0	12	0	0	0	0
NC	10	0	0	0	0	0	0	0
Total	148	164			58			19
	17.46%	19.34%			6.84%			2.24%

BLV=Bovine leukemia virus, BVDV=Bovine viral diarrhea virus, BoHV-1=Bovine alphaherpesvirus 1, NC=*Neospora caninum*. I=Seropositivity with one infectious agent, I=Seropositivity with two infectious agents in the same animal, III=Seropositivity with three infectious agents in the same animal, IV=Seropositivity with four infectious agents in the same animal

Bivariate statistical analysis (Table-2) with BLV seroprevalence as the outcome showed that variables such as herd, breed, gender, and age were statistically associated with BLV (p < 0.05). The bovine infectious abortion agents BVDV, BoHV-1, and NC were statistically associated with BLV seroprevalence (p < 0.05). The mixed-effects logistic regression model did not show a herd random effect. All variables of the bivariate analysis were entered into the multivariate logistic regression analysis for BLV. Table-3 shows the multivariate logistic regression model of the variables associated with BLV seroprevalence. Through a backward elimination process in the final model, seropositivity of BVDV (OR: 10.8; 95% confidence interval [CI]: 7.5–15.6) and seropositivity of bovine α-herpesvirus-1 – BoHV-1 (OR 1.8; 95% CI: 1.1–3.0) are associated with the seropositivity of BLV (Table-3). Regarding breed, the odds of BLV seroprevalence are lower in Romosimuano (ROMO) compared with CCC.

**Table-2:** Bivariate statistical analysis of the variables associated (p = 0.15) with the seroprevalence of bovine leukemia virus in grazing Creole cows (n = 848) from two tropical herds in the Colombian Caribbean.

Variable	OR	Standard error	p-value	95% CI	

				LL	UL
Herd					
AGB	Ref.				
GIP	0.35	0.11	<0.001	0.18	0.66
Breed					
CCC	Ref.				
ROMO	0.31	0.04	<0.001	0.22	0.42
Gender					
M	Ref.				
F	2.50	0.44	<0.001	1.76	3.54
Age					
<1 year	Ref.				
≥1 year	1.44	0.24	0.032	1.03	2.02
BVDV					
Seronegative	Ref.				
Seropositive	12.60	2.22	<0.001	8.92	17.81
BoHPV-1					
Seronegative	Ref.				
Seropositive	3.5	0.75	<0.001	2.36	5.40
NC					
Seronegative	Ref.				
Seropositive	2.9	0.71	<0.001	1.82	4.73

LL=Lower limit, UL=Upper limit, CI=Confidence interval, OR=Odds ratio, BVDV=Bovine viral diarrhea virus, BoHV-1=Bovine α-herpesvirus-1, NC=*Neospora caninum*, GIP=Genetic improvement program herd, AGB=Animal Germplasm Bank herd, CCC=Costeño con Cuernos, ROMO=Romosinuano, M=Male, F=Female

**Table-3:** Multivariate logistic regression model of the variables associated with the seroprevalence of bovine leukemia virus in grazing Creole cows (n = 848) from two tropical herds in the Colombian Caribbean.

Variable	OR	Standard error	p-value	95% CI	

				LL	UL
Intercept	0.23	0.03	<0.001	0.17	0.31
BVDV			<0.001*		
Seronegative	Ref.				
Seropositive	10.86	2.00	<0.001	7.56	15.60
BoHV-1			<0.001*		
Seronegative	Ref.				
Seropositive	1.87	0.47	0.012	1.14	3.07
Breed			<0.001*		
CCC	Ref.				
ROMO	0.33	0.06	0.000	0.22	0.48

* Global p-value for the entire categorical variable, LL=Lower limit, UL=Upper limit, CI=Confidence interval, OR=Odds ratio, BVDV=Bovine viral diarrhea virus, BoHV-1=Bovine α-herpesvirus-1, NC=*Neospora caninum*, CCC=Costeño con Cuernos, ROMO=Romosinuano

## Discussion

This study adds to the knowledge of infectious diseases among Colombian Caribbean Creole cattle. The analysis and comparison of infectious problems in non-Creole bovine breeds from tropical herds are crucial because the studied infectious agents are endemic to all bovine breeds in Colombia.

The ROMO and CCC breeds represent an important genetic heritage of Colombia, and AGROSAVIA maintains a national genetic conservation program. These Creole breeds are known for their rusticity and adaptation to the climatic conditions of the low tropics. However, despite the lack of quantified indicators, field observations of the cattle herd suggest an increase in animals with low body conditions and reproductive problems. The health management of these animals is currently based on a preventive vaccination scheme for officially controlled diseases, such as foot-and-mouth disease, rabies, and brucellosis, as well as other self-controlled enzootic infectious diseases like clostridium (*Clostridium* spp.) and *Pasteurella* (*Pasteurella* spp.). However, health management for reproductive infectious disorders is not common.

In the bivariate analysis, all factors showed a significant correlation with BLV seroprevalence. Multivariate statistics did not reveal a link between age and cattle herd type with the integrated variables. Age and cattle herd type remain crucial factors to consider when designing preventive health plans against infectious agents. BLV had a significant association with the variable breed. Animals of the CCC breed had a greater risk of infection. This finding is consistent with previous reports by Hernández Herrera *et al*. [10] on other Colombian Creole breeds. The results also revealed that the CCC breed had a higher seroprevalence for BLV than the ROMO breed. Colombian Creole cattle have high accumulated frequencies of bovine leukocyte antigen (BoLA-DRB3.2) alleles that provide resistance to BLV infection [9, 10]. Another study evaluated BLV seroprevalence in eight Colombian Creole breeds, where 30 animals of each racial group were tested, and variations from 0.0% to 83.3% (average of 26.7%) were found. The ROMO breed had 0.0% seroprevalence, whereas the CCC breed had 23% [10].

Various studies [11–16] have determined that BLV, BVDV, BoHV-1, and NC are endemic microorganisms in Colombia. For instance, a study that used polymerase chain reaction (PCR) to diagnose BLV in 289 cows from 75 cattle farms found that the animal prevalence was 62%, whereas the farm-level prevalence was 92% [11]. Another investigation that analyzed the seroprevalence of the four infectious agents in 440 bovine samples obtained the following results: BLV (21.8%), BVDV (29.7%), BoHV-1 (48.2%), and NC (63%) [12]. In addition, a different study determined the antigenic circulation of NC in bovine females, revealing seropositivity of 10.2%, and a considerable number of cows experienced reproductive problems, such as abortions (11%), fetal mummification (20%), and repeated heat (10%) [13]. Recently, a study that analyzed 840 samples from cows across various regions of the country found the following seropositivity: BLV (14.64%), BVDV (25.83%), and NC (19.29%) [14].

BLV infection in animals may result in immunosuppression, thereby increasing the likelihood of secondary infections from other pathogens such as BVDV, BoHV-1, and NC [5, 15, 16]. Immunosuppression can also leave infected animals vulnerable to pathogen-induced abortions. Directly, BLV can cause transplacental transmission in cows, as evidenced by a study in which 16.25% of aborted fetuses tested positive for BLV through PCR testing [17].

Control of BLV infection in ruminants is challenging due to its integration into B cells and the host genome, ensuring long-term infection. The infection often spreads without symptoms. Only 1%–5% of BLV-infected animals develop an aggressive lymphoma known as enzootic bovine leukosis (EBL) following a long latency period [18]. Managing BLV infection in cattle poses a substantial challenge due to various aspects.

The reduction in both total and specific plasma immunoglobulin M levels in cows infected with BLV can potentially be explained by interference in the gene expression pathway [19]. This finding is further supported by the connection of BLV miRNAs with the target genes’ expression in infected animals [19–21]. The high prevalence of BLV infection, combined with its long symptomless period and immunosuppressive effect, emphasizes the need for effective control measures. Therefore, implementing and prioritizing healthy protocols against BLV are crucial for improving the sanitary status of cattle herds. In addition, it is crucial to consider immunosuppression and the increased risk of secondary infections caused by BVDV [6]. Furthermore, the immunosuppressive effects of BLV- BVDV coinfections can synergistically impact cattle, emphasizing the urgent need for preventive measures to mitigate the health risks associated with these infectious agents.

This study was designed as a retrospective cross-sectional study, which limits the ability to confirm that BLV is the primary infection. Nonetheless, the findings indicate that BLV is one of the most prevalent microorganisms in the studied animal population. In addition, most animals seropositive for the other infectious agents were also seropositive for BLV, demonstrating a statistical association with all of them in the univariate analysis. These results are consistent with those of other studies that have highlighted the effect of BLV on the risk of secondary infections [5, 17, 22].

The univariate analysis found a significant association between BLV and NC, but this was not maintained in the final model. Similar behavior was observed between BLV and BoHV-1. Although one study found an association between NC and BLV-seropositive cows [16], another study did not identify an association with BoHV-1 [23]. Dairy cows were diagnosed with BLV, BoHV-1, and BVDV, and a significant association was found between them [15]. Previous studies in Colombia evaluated the serological status of BLV, BVDV, BoHV-1, and NC, but they did not analyze the association between BLV and abortion infectious agents [12, 14].

This study revealed that the two microorganisms with the highest seroprevalence were BLV (30.78%) and BVDV (33.01%). These microorganisms also showed significant associations in the final multivariate logistic regression model. This highlights that BVDV-seropositive animals are almost 11 times more likely to be BLV-seropositive. The relationship between BLV and BVDV was reported in another study as well [15], while some authors have yet to identify it [24], and other authors have not investigated it due to a lack of epidemiological interest [12, 14].

Preventive treatments may represent the most effective control strategy for BLV and BVDV. In a study, vaccination with a live attenuated virus against BVDV in cows naturally co-infected with BLV-induced antibody production, suggesting that the immunosuppressive effect did not hinder seroconversion elicited by the vaccine, except in animals persistently infected with BLV [25]. However, this approach only enables the control of BVDV infection. A recent vaccine developed for the preventive treatment of BLV showed efficacy and safety in herd conditions [26]. However, despite advancements in the investigation of experimental vaccines, a commercially available vaccine for the control of EBL is not yet available [3].

The current study highlights a complex epidemiological situation, as BLV lacks pharmacological treatment, and a preventive vaccine is not yet available. Moreover, while there is a vaccine for BVDV, its effectiveness is compromised by persistently infected animals that continuously shed high levels of viral particles [25, 27]. The high prevalence of BLV and BVDV in cattle herds requires swift, strong, and decisive sanitary interventions to significantly reduce the impact on herd productivity indicators and mitigate the high risk of secondary infectious agents. Management practices, such as rectal palpation, artificial insemination, and injection administration, have been identified as risk factors for BLV incidence in adult animals [28], particularly in cases of persistent lymphocytosis or high proviral load [29]. Control strategies should guide farmers in implementing biosecurity measures and reducing exposure risks to infected lymphocytes during livestock management activities.

## Conclusion

In developing countries like Colombia, the existence of high seroprevalence rates for antibodies against BLV, BVDV, BoHV-1, and NC presents significant health and economic challenges. The co-infection of BLV and BVDV in herds is especially hazardous to cattle health. Implementing preventive measures against BLV and BVDV will help control their spread and enhance herd productivity and economic indicators by improving sanitary conditions. Establishing control programs is essential to halt the progression of these pathogens. Implementation of good sanitary practices and periodic monitoring in infected herds are crucial to minimize incidence rates by identifying, isolating, or culling positive BLV or BVDV animals. This study was designed as a retrospective cross-sectional study, which limits the ability to confirm that BLV is the primary infection. Further studies to confirm the primary infection of BLV with an active viral coinfection are necessary and the factors associated with these phenomena.

## Authors’ Contributions

MOP, MDR, and TOS: Designed the study. MDR: Collected samples and performed serological analysis. MOP, TOS, and SM: Supervised the study. MOP and DVT: Performed the statistical analysis. MOP, MDR, TOS, SM, and DVT: Drafted and edited the manuscript. SM: Provided conceptualization and technical assistance. All authors have read, reviewed, and approved the final manuscript.

## References

[1] Marawan M, Alouffi A, El-Tokhy S, Badawy S, Shirani I, Dawood A, Guo A, Almutairi M.M, Alshammari F.A, Selim A (2021). Bovine leukaemia virus:Current epidemiological circumstance and future prospective. Viruses.

[2] Delarmelina E, Buzelin M.A, de Souza B.S, Souto F.M, Bicalho J.M, Câmara R.J.F, Resende C.F, Bueno B.L, Victor R.M, Galinari G.C.F, Nunes C.B, Leite R.C, Costa É.A, Reis J.K.P.D (2020). High positivity values for bovine leukemia virus in human breast cancer cases from Minas Gerais, Brazil. PLoS One.

[3] World Organization for Animal Health (WOAH) (2023). Enzootic bovine leukosis. Manual of Diagnostic Tests and Vaccines for Terrestrial Animals.

[4] Lv G, Wang H, Wang J, Lian S, Wu R (2021). Effect of BLV infection on the immune function of polymorphonuclear neutrophil in dairy cows. Front. Vet. Sci.

[5] Frie M.C, Coussens P.M (2015). Bovine leukemia virus:A major silent threat to proper immune responses in cattle. Vet. Immunol. Immunopathol.

[6] Al-Kubati A.A.G, Hussen J, Kandeel M, Al-Mubarak A.I.A, Hemida M (2021). Recent advances on the bovine viral diarrhea virus molecular pathogenesis, immune response, and vaccines development. Front. Vet. Sci.

[7] Dohoo I.R, Martin W, Stryhn H.E (2009). Veterinary Epidemiologic Research.

[8] Corredor-Figueroa A.P, Salas S, Olaya-Galán N.N, Quintero J.S, Fajardo A, Soñora M, Moreno P, Cristina J, Sánchez A, Tobón J, Ortiz D, Gutiérrez M.F (2020). Prevalence and molecular epidemiology of bovine leukemia virus in Colombian cattle. Infect. Genet. Evol.

[9] Dean A.G, Sullivan K.M, Soe M.M (2013). OpenEpi:Open-Source Epidemiologic Statistics for Public Health, Version 3.0. https://www.openepi.com.

[10] Hernández Herrera D.Y, Posso Terranova A.M, Benavides J.A, Muñoz Flórez J.E, Giovambattista G, Álvarez-Franco L.A (2011). Bovine leukemia virus detection in Creole Colombian breeds using nested-PCR [Detección del virus de la leucosis bovina en ganado criollo Colombiano mediante PCR-anidado]. Acta Agronómica.

[11] Hernández-Herrera D.Y, Muñoz Flórez J.E, Álvarez-Franco L.A (2014). Association between the locus BOLA-DRB3.2 and bovine leukemia virus in creole colombian breeds [Asociación del locus BOLA-DRB3.2 con el virus de la leucosis bovina en el ganado criollo Colombiano]. Rev. Colomb. Cien. Anim.

[12] Vargas-Niño A, Vargas R.J, Parra-Martin J.A, Vásquez R.M, Góngora O.A, Mogollón-Waltero E (2018). Serological status of IBR, BVD, leucosis, Leptospira and Neospora caninum in bovine females of the department of Santander, Colombia [Estado serológico para IBR, DVB, Leucosis, *Leptospir*a y *Neospora caninu*m en hembras bovinas del Departamento de Santander, Colombia]. Rev. MVZ Córdoba.

[13] Oviedo S.T, Betancur H.C, Mestra P.A, González T.M, Reza G.L, Calonge G.K (2007). Serological study about neosporosis in cattle with reproductive disorders in Monteria, Cordoba, Colombia [Estudio serológico sobre neosporosis en bovinos con problemas reproductivos en Montería, Córdoba, Colombia]. Rev. MVZ Córdoba.

[14] Naranjo Guerrero L.F, Rodríguez Colorado N, Mejía Araque J (2022). Prevalence of bovine viral diarrhea, bovine neosporosis, enzootic bovine leukosis and bovine paratuberculosis in dual-purpose cows in conditions of the Colombian tropics [Prevalencia de diarrea viral bovina, neosporosis bovina, leucosis bovina enzoótica y paratuberculosis bovina en vacas de doble propósito en condiciones del trópico Colombiano]. Rev. Investig. Vet. Perú.

[15] Bilge-Dagalp S, Can-Sahna K, Yıldırım Y, Karaoğlu T, Alkan F, Burgu I (2008). Effects of bovine leucosis virus (BLV) infection on the bovine viral diarrhoea virus (BVDV) and bovine herpes virus 1 (BHV1) seroprevalences in dairy herds in Turkey. Rev. Méd. Vét.

[16] Vanleeuwen J.A, Haddad J.P, Dohoo I.R, Keefe G.P, Tiwari A, Scott H.M (2010). Risk factors associated with *Neospora caninum* seropositivity in randomly sampled Canadian dairy cows and herds. Prev. Vet. Med.

[17] Montanari K.C.S, Fusuma M.M, Lacerda A.M.D, Okuda L.H, Pituco E.M, de Carvalho A.F, Castro V, Piatti R.M, Pinheiro E.S, Harakava R, Del Fava C (2019). Bovine leukemia virus in bovine aborted fetuses. J. Leuk.

[18] Okagawa T, Shimakura H, Konnai S, Sait o M, Matsudaira T, Nao N, Yamada S, Murakami K, Maekawa N, Murata S, Ohashi K (2022). Diagnosis and early prediction of lymphoma using high-throughput clonality analysis of bovine leukemia virus-infected cells. Microbiol. Spectr.

[19] Frie M.C, Droscha C.J, Greenlick A.E, Coussens P.M (2018). MicroRNAs encoded by Bovine Leukemia Virus (BLV) are associated with reduced expression of B cell transcriptional regulators in dairy cattle naturally infected with BLV. Front. Vet. Sci.

[20] Ma H, Lippolis J.D, Casas E (2022). Expression profiles and interaction of MicroRNA and transcripts in response to bovine leukemia virus exposure. Front. Vet. Sci.

[21] Mousavi M, Fasaei B.N, Tafsiri E, Rayat R.Y, Langeroudi A.G (2022). Investigation of the target genes of BLV miRNAs and the expression levels of miR-B4-3p and miR-B2-5p in cattle infected with Bovine Leukemia Virus. Vet. Res. Forum.

[22] Cuesta L.M, Liron J.P, Nieto Farias M.V, Dolcini G.L, Ceriani M.C (2020). Effect of bovine leukemia virus (BLV) infection on bovine mammary epithelial cells RNA-seq transcriptome profile. PLoS One.

[23] Juliarena M.A, Poli M, Ceriani C, Sala L, Rodríguez E, Gutierrez S, Dolcini G, Odeon A, Esteban E.N (2009). Antibody response against three widespread bovine viruses is not impaired in Holstein cattle carrying bovine leukocyte antigen DRB3.2 alleles associated with bovine leukemia virus resistance. J. Dairy Sci.

[24] Nikbakht G, Tabatabaei S, Lotfollahzadeh S, Fasaei B.N, Bahonar A, Khormali M (2015). Seroprevalence of bovine viral diarrhoea virus, bovine herpesvirus 1 and bovine leukaemia virus in Iranian cattle and associations among studied agents. J. Appl. Anim. Res.

[25] Pichardo-Matamoros D, Elizondo-Salazar J, Jiménez-Sánchez C (2020). Seroconversion to BVDV in cows infected with CLABE and HVB1:Basis of colostrogenesis and effect of persistent infection caused by BVDV [Seroconversión al VDVB en vacas coinfectadas con VLBE y HVB1:Fundamento de la calostrogénesis y efecto de la infección persistente causada por el VDVB]. Arch. Latinoam. Prod. Anim.

[26] Suárez Archilla G, Gutiérrez G, Camussone C, Calvinho L, Abdala A, Alvarez I, Petersen M, Franco L, Destefano G, Monti G, Jacques J.R, Joris xT, Willems L, Trono K (2022). A safe and effective vaccine against bovine leukemia virus. Front. Immunol.

[27] Khodakaram-Tafti A, Farjanikish G.H (2017). Persistent bovine viral diarrhea virus (BVDV) infection in cattle herds. Iran. J. Vet. Res.

[28] Benavides B, Monti G (2022). Assessment of natural transmission of bovine leukemia virus in dairies from Southern Chile. Animals (Basel).

[29] Ruggiero V.J, Norby B, Benitez O.J, Hutchinson xH, Sporer K.R.B, Droscha C, Swenson C.L, Bartlett P.C (2019). Controlling bovine leukemia virus in dairy herds by identifying and removing cows with the highest proviral load and lymphocyte counts. J. Dairy Sci.

